# The influence of soil organic matter content on the toxicity of pesticides to the springtail *Folsomia candida*

**DOI:** 10.1093/etojnl/vgae048

**Published:** 2025-01-06

**Authors:** Bart G van Hall, Christopher J Sweeney, Melanie Bottoms, Cornelis A M van Gestel

**Affiliations:** Faculty of Science, Amsterdam Institute for Life and Environment (A-LIFE), Vrije Universiteit Amsterdam, Amsterdam, The Netherlands; Product Safety, Syngenta—Jealott’s Hill International Research Centre, Bracknell, United Kingdom; Product Safety, Syngenta—Jealott’s Hill International Research Centre, Bracknell, United Kingdom; Faculty of Science, Amsterdam Institute for Life and Environment (A-LIFE), Vrije Universiteit Amsterdam, Amsterdam, The Netherlands

**Keywords:** environmental risk assessment, correction factor, soil properties, soil invertebrates, exposure route

## Abstract

The European environmental risk assessment (ERA) of pesticides to soil invertebrates applies a correction factor (CF) of 2 to endpoints derived from toxicity tests with lipophilic pesticides (log K_ow_ > 2) to correct for differences in organic matter (OM) content between artificial soil (AS) and natural soils. Because the applicability of this CF to springtails has never been assessed, this study investigated the influence of soil OM content on the toxicity of five pesticide active substances differing in lipophilicity to the springtail *Folsomia candida*. Toxicity tests following Organisation for Economic Cooperation and Development guideline 232 were conducted in AS containing 10%, 5%, and 2.5% peat and a natural soil (LUFA 2.2) with 4.5% OM. For all pesticides, toxicity (median lethal and median effect concentrations [LC50s, EC50s]) differed significantly between soils and strongly negatively correlated with soil OM content in AS (*r*^2^ > 0.88). Utilizing the regression equations derived from the data, LC50s and EC50s were calculated for soils with 10% and 5% OM. For EC50s, the differences in model-estimated toxicity between these soils ranged from 1.85 to 3.23, sometimes exceeding the CF of 2. To identify differences between species, data from a sister paper on the earthworm *Eisenia andrei* was used. Although ratios between model-estimated EC50s in soils containing 10% and 5% OM were similar between species (2.08–3.24 for earthworms), pesticide-specific toxicity–OM relationships differed in some cases. Non-lipophilic pesticides were influenced by soil OM content in a similar manner as the lipophilic pesticides, showing that the influence of soil properties on non-lipophilic pesticides may currently be overlooked. Overall, this study shows that the CF of 2 suffers from erroneous assumptions concerning lipophilicity, OM content, and toxicity. Further research is required to improve our mechanistic understanding of the relationship between toxicity and soil OM content, ultimately increasing the ecological relevance of CFs used in ERAs.

## Introduction

In Europe, the environmental risk assessment (ERA) of pesticides to soil invertebrates follows a tiered approach. In the first tier, standardized laboratory toxicity tests are performed, in which test organisms are exposed to the test compound in artificial soil (AS) under standardized conditions. Endpoints such as the median lethal concentration (LC50) or the 50% and 10% effect concentrations (EC50 and EC10) from these tests are used to calculate a toxicity-to-exposure ratio (TER) by dividing them with the predicted environmental concentration (PEC) of the pesticide under the intended use in the field. When the TER > 5, the PEC is sufficiently far below the concentrations at which an effect is observed, and no risk for soil organisms living in the field is assumed. On the other hand, when the TER < 5, the precautionary principle is applied, meaning that risk at the lower tier is stated and the ERA can be moved to a higher tier.

Historically, the first laboratory toxicity tests on soil invertebrates focused on earthworm (*Eisenia andrei* or *Eisenia fetida*) mortality in AS containing 10% sphagnum peat, after 14 days exposure following Organisation for Economic Cooperation and Development (OECD) test guideline 207 (OECD, 1984). The use of AS was preferred over natural soils because the materials are readily available and create a standardized test system, facilitating a higher degree of comparability between laboratories and substances. In the late 1980s, Van Gestel and Ma (1988) and Van Gestel and Van Dis (1988) showed that the toxicity of lipophilic organic chemicals (log K_ow_ > 2) could significantly differ between soils, showing reduced toxicity in soils having higher organic matter (OM) contents. This work led to the inclusion of an additional correction factor (CF) of 2 within the ERA to compensate for the differences in toxicity between AS and agricultural soils due to differences in OM content (European and Mediterranean Plant Protection Organization, 2003; Santé et Consommateurs, 2002). This CF of 2 has been adopted outside of Europe as well, for instance in Australia (Australian Pesticides and Veterinary Medicines Authority, 2019) and the Andean states (Comunidad Andina, 2019).

Since the development of these test systems using earthworms, new laboratory toxicity tests have been introduced using test organisms such as springtails (OECD 232, 2016a), predatory mites (OECD 226, 2016b), and enchytraeids (OECD 220, 2016c). These are generally conducted in AS containing 5% instead of 10% peat. Similarly, the focus of the soil invertebrate ERA has shifted from lethal to more sensitive sublethal endpoints such as reproduction (OECD 222, 2016d). Despite these significant changes in the first tier of ERA, the suitability of the CF of 2 to chronic endpoints calculated on organisms other than earthworms has never been assessed but is applied.

Van Hall et al. (2024) investigated whether the additional CF of 2 is suitable for sublethal effects on earthworms. They exposed earthworms (*E. andrei*) to five pesticides with different lipophilicity in three ASs and a natural reference soil. The obtained toxicity–OM relationships differed between pesticides, lethal, and sublethal endpoints, and soil OM content also influenced the toxicity of non-lipophilic pesticides. Consequently, they concluded that the CF of 2 may not be an appropriate factor to correct for differences in toxicity between AS and natural soils, because the fundamentals on which it is based (i.e., relationships between pesticide lipophilicity, OM content, and toxicity) were incorrect.

In a literature review by the same authors, the influence of soil OM content on pesticide toxicity to soil invertebrates was investigated, and the question as to whether soil OM content influenced pesticide toxicity to multiple soil invertebrate groups in the same manner was addressed (Van Hall et al., 2023). The authors reported similar findings as Van Hall et al. (2024) and illustrated this, for instance, with differences in toxicity–OM relationships between enchytraeids and springtails exposed to the herbicide phenmedipham, possibly related to differences in exposure routes. However, they concluded that there were insufficient data available in the literature from which to derive robust conclusions. As such, it remains unclear if the additional CF of 2 is appropriate for use across different invertebrate groups.

The aim of this study was to assess if the additional CF of 2, applied in the ERA for lipophilic pesticides, is appropriate for springtails. To do this, the influence of soil OM content on pesticide toxicity to the springtail *Folsomia candida* was investigated by performing standardized toxicity tests using multiple soils and pesticides (active substances) varying in lipophilicity. The main questions were: (1) Can toxicity–OM relationships be quantified for springtails, and do they support the application of the CF of 2? (2) Are the toxicity–OM relationships similar for springtails and earthworms? (3) Can the toxicity–OM relationships be used to predict toxicity in natural soils? And (4) Does pesticide lipophilicity drive toxicity–OM relationships? The second and third questions were answered using data from Van Hall et al. (2024) on a similar study with earthworms.

## Materials and methods

### Test organisms, soils, and pesticides

Springtails (*F. candida*) originated from a laboratory culture at the Amsterdam Institute for Life and Environment (A-LIFE), Vrije Universiteit Amsterdam, The Netherlands. Animals were cultured on moist plaster of Paris mixed with charcoal (10% w/w) in a climate chamber set at 16 ± 1 °C, 75% relative humidity, a 16:8-hr light:dark photoperiod and were richly fed with dry baker’s yeast (AB Mauri, The Netherlands). The springtails were age-synchronized by allowing 30 adults to lay eggs onto freshly prepared plaster of Paris for 2 days, after which they were removed, and the eggs hatched in the following 8–10 days. The juveniles were fed dry baker’s yeast twice a week until they were 10–12 days old and were used in the toxicity tests.

Test soils and pesticides were identical to those used in the earthworm toxicity tests described by Van Hall et al. (2024). In short, tests were conducted using three OECD ASs with different proportions of sphagnum peat, kaolin clay, and quartz sand, as well as a natural LUFA 2.2 soil (LUFA Speyer, Germany). The ASs were created by combining dried finely ground sphagnum peat (10%, 5%, 2.5%; HAWITA Gruppe GmbH, Germany), kaolin clay (20%; Keramikos, The Netherlands), quartz sand with a grain size of 0.1–0.3 mm (70%, 75%, 77.5%; Multiquartz, The Netherlands), and CaCO_3_ (0.1%–0.5%; Merck, Germany) to adjust the pH (0.01 M CaCl_2_) of the soils to 6.0 ± 0.5. The LUFA 2.2 soil was dried at 60°C on arrival and stored in complete darkness at room temperature until further use. Prior to the toxicity tests, the ASs were moistened to 25% of their maximum water holding capacity (WHC_max_) for a period of 4–6 days to allow the acidity levels to stabilize. The characterization of the test soils is further described in Van Hall et al. (2024), and the soil properties are shown in Table 1.

**Table 1.** Properties of the artificial and natural soils used to determine the influence of soil organic matter content on pesticide toxicity to *Folsomia candida*.

Soil properties	OECD 10%	OECD 5%	OECD 2.5%	LUFA 2.2
Sphagnum peat (%)	10	5	2.5	–
Organic matter (%)^a^	9.8 ± 1.1	6.2 ± 0.5	3.6 ± 0.9	4.5 ± 0.1
(Kaolin) clay (%)	20	20	20	10.8 ± 1.7^b^
(Quartz) sand (%)	70	75	77.5	72.3^b^
pH (0.01 M CaCl_2_)^c^	5.97 ± 0.05	5.81 ± 0.05	5.89 ± 0.08	5.54 ± 0.03
WHC_max_ (%)^d^	55 ± 2.5	39.8 ± 1.3	32.1 ± 1.2	48.9 ± 1.2

a Average ± SD of controls at the start of the test (*n *=* *6). No measurements were taken for the imidacloprid and carbendazim experiments due to a lack of soil samples.

b Mean values of different batch analyses provided by LUFA Speyer. Clay particles are <0.002 mm, sand particles 0.063–2.0 mm.

c Average ± SD of lowest test concentrations at start of the test (*n *=* *8). Lowest test concentrations were used because not enough control soil samples were available. No measurements were taken for the carbendazim experiments.

d WHC_max_ was determined following OECD guideline 232, Annex 5 (OECD, 2016a). Average ± SD of artificial soils (*n *=* *6) and LUFA 2.2 soil (*n *=* *2). Samples were taken from the soils prepared in bulk.

*Note*. OECD 10%: artificial soil containing 10% sphagnum peat; OECD 5%: artificial soil containing 5% sphagnum peat; OECD 2.5%: artificial soil containing 2.5% sphagnum peat; LUFA 2.2: natural reference soil obtained from Landwirtschaftliche Untersuchungs- und Forschungsanstalt; WHC_max_: Maximum water holding capacity of the test soil.

The selected active substances were: chlorpyrifos, lindane, cyproconazole, carbendazim, and imidacloprid. These pesticides vary in their lipophilicity, and their half-lives (DT50) are sufficiently long to ensure that degradation does not play a significant role during the toxicity tests. An overview of the suppliers, chemical purity and chemical properties are shown in Table 2, concentration ranges chosen for the toxicity tests in the different soils are shown in online supplementary material Table S1.

**Table 2.** Suppliers, purity and chemical properties of the pesticides used in the springtail toxicity tests in different soils.^a^

Pesticide	Manufacturer	Purity	Log K_ow_	DT50 (days)	pK_a_	Water solubility (mg L^–1^)
Chlorpyrifos	Dr. Ehrenstorfer	99.6%	4.7	386	–	1.05
Lindane	Sigma Aldrich	98.0%	3.5	980	–	8.52
Cyproconazole	Syngenta	99.7%	3.1	142	–	93
Carbendazim	Sigma Aldrich	97%	1.5	40	4.2	8
Imidacloprid	Bayer	98%	0.57	191	–	610

a Data were obtained from the Pesticide Properties Database (Lewis et al., 2016).

*Note.* DT50: time required for 50% degradation of the pesticide; pK_a_: acid dissociation constant of the pesticide.

### Soil spiking and toxicity tests

Acetone (99.8%, purity; VWR Chemicals, The Netherlands) was used as a carrier solvent for chlorpyrifos, lindane, cyproconazole, and carbendazim, and demineralized water for imidacloprid. When acetone was used, pesticide solutions were added to 10% (w/w) of the total mass of soil needed for a treatment in such a way that the soil sample was covered with approximately 1 mm of pesticide solution. Solvent controls (SCs) were included when acetone was used as a carrier solvent. The containers were closed and left for 6 hr to allow for partitioning of the pesticide in the soil, after which they were opened in a fume hood to evaporate the acetone. The following day, the spiked soil was added to the remaining 90% of the soil and mixed homogenously using a household mixer. Demineralized water was added to adjust the soil moisture content to 50% ± 5% of the WHC_max_. Soils were then mixed once more and divided over the replicates. For imidacloprid, the pesticide solutions were added to achieve 50% ± 5% of WHC_max_ directly.

Toxicity tests were conducted in accordance with OECD guideline 232 (OECD, 2016a). At the start of the tests, 10 age-synchronized animals were placed in 100 ml glass jars containing 30 g of moist (spiked) test soil (six replicates). The test jars were randomly distributed in a climate-controlled room set at 20 ± 1°C, with a 16:8-hr light:dark photoperiod, and a relative air humidity of 75%. At the start of the test and each subsequent week, demineralized water was added to replenish any moisture loss, and a few grains of dry baker’s yeast were provided for food. After 28 days, one of the replicates was used to measure pH and pesticide concentrations. For the other replicates, approximately 100 ml of water was added to the test jars, and the soil/water mixture was transferred into a plastic beaker. The mixture in the beaker was gently stirred with a spatula to allow the floatation of the animals. For the ASs, 2–3 drops of Indian ink were added to increase the contrast between the springtails and the background. The floating animals were photographed using a Nikon^®^ digital camera (model COOLPIX P510). Adults and juveniles were counted manually using ImageJ (Ver. 1.53) and the quality criteria from OECD guideline 232 were used to assess the validity of the toxicity tests. In short, average survival and juveniles produced in the controls should be ≥80% and ≥100, respectively, and the coefficient of variation for reproduction should be ≤30%.

### Chemical analysis

For each treatment group, soil samples (approximately 20 g; *n* = 2) were collected at the start and end of the tests and stored at –20°C. The soil samples were used to measure soil pH, to determine the accuracy of the soil spiking, and to assess pesticide concentration over the test period. After all tests were performed, pesticide concentrations were measured in the SC (control for imidacloprid) at the start of the tests, and in the concentrations around the EC50 values at the start and end of the tests. Soils were analyzed by the company Normec Groen Agro Control (Delfgauw, The Netherlands) following the Quick, Easy, Cheap, Effective, Rugged, and Safe method using gas chromatography–mass spectrometry and liquid chromatography–tandem mass spectrometry, and applying certified chemical analytical procedures and quality control measures. Pesticide extractions were done using exhaustive measures. Carbendazim concentrations were not quantified, because no effects on springtail reproduction were observed, and because of budgetary constraints; results from a sister paper on earthworms using the same spiking method and soils showed good recoveries also for carbendazim (Van Hall et al., 2024). The detection limit was 0.01 mg kg^−1 ^dry soil. Because the pesticide concentrations were quantified in moist soils at 50% WHC_max_, the measured values were corrected for moisture content to give mg kg^−1 ^dry soil values before data analysis.

### Data analysis

All statistical analyses were performed in RStudio Ver. 2023.12.0 running R Ver. 4.2.1 (R Core Team, 2021). Levene’s tests were used to assess the homogeneity of variance of the data. Unpaired Student *t*-tests were performed to identify if survival and reproduction differed between control and SC groups. If no significant differences were observed, controls and SCs were grouped together for the data analysis. Dose–response curves for effects on survival and reproduction were fit using the drc package applying a general three parameter dose–response model (Ritz et al., 2015). These models were used to estimate EC10, EC50, and LC50 values and corresponding 95% confidence intervals (CIs). To identify differences in toxicity between the soils for each test compound, a likelihood-ratio test was performed comparing the models for each soil with a constrained model (constraining the models for each soil to the same EC50 or LC50; Zhang et al., 2019). Toxicity–OM relationships were quantified for the ASs through linear regression analyses. To determine if the CF of 2 is appropriate for springtails, the regression equations were used to model the toxicity in soils containing 10.0% and 5.0% soil OM, and the ratios between these model-estimated toxicity values were compared to the CF of 2. To determine if toxicity–OM relationships were driven by pesticide lipophilicity, model-estimated toxicity ratios for LC50 and EC_x_ values were compared between the pesticides.

## Results

Soil pH measured at the lowest and highest test concentrations remained constant (± 0.6 pH units) over the course of the tests, and pesticide addition did not change soil pH (± 0.2 pH units; online supplementary material Table S2). Nominal and measured pesticide concentrations in the test soils are shown in the online supplementary material Table S3, and average pesticide recovery is presented in Table 3. All SC soils were uncontaminated. Average recovery at the start of the tests differed between pesticides and ranged between 82.2% and 150%, but recovery did not differ much between the four soils for each pesticide. After 28 days of exposure, pesticide recoveries generally were similar, being 0%–23% lower than at the start, with the exception of chlorpyrifos in LUFA 2.2 soil, where an approximate 50% loss was observed. Because pesticide recovery at the start was similar between soils for each pesticide and variations in recovery between concentrations may also be due to soil heterogeneity, the average pesticide recovery at the start of the experiments was used to correct the nominal concentrations. The corrected nominal concentrations were then used in the data analysis.

**Table 3.** Average pesticide recovery (%) in artificial and natural springtail toxicity test soils after 0 and 28 days.^a^

Pesticide	Day	OECD 10%	OECD 5%	OECD 2.5%	LUFA 2.2	Average
Chlorpyrifos	0	89.3	83.9	69.6	86.1 ± 17.4	82.2 ± 5.0
	28	76.5	95.9	69.6	43.1 ± 8.7	71.3 ± 21.9
Lindane	0	153	150	153 ± 9.0	143 ± 5.6	150 ± 4.4
	28	133	115	129 ± 1.2	123 ± 7.0	125 ± 7.6
Cyproconazole	0	81.3	92.9 ± 0.1	83.1 ± 1.2	98.4 ± 13.9	89.0 ± 8.1
	28	81.9	81.3 ± 1.7	81.4 ± 3.0	97.4 ± 4.2	85.5 ± 8.0
Imidacloprid	0	102 ± 19.0	112 ± 5.1	104 ± 0.7	104 ± 0.8	105.2 ± 4.4
	28	109 ± 24.4	99.0 ± 12.7	93.0 ± 11.8	79.8 ± 0.0	95.2 ± 12.3

a Carbendazim concentrations were not quantified because no toxic effects on springtails were observed. Soil Pesticide concentrations were quantified in spiked soils from vessels containing organisms, at concentrations around the median effective concentration (EC50) values. Values are shown ± SD (*n *=* *1 When no SD is given, *n *=* *2 for all other individual soils, *n *=* *4 for average over all soils together).

*Note*. OECD 10%: artificial soil containing 10% sphagnum peat; OECD 5%: artificial soil containing 5% sphagnum peat; OECD 2.5%: artificial soil containing 2.5% sphagnum peat; LUFA 2.2: natural reference soil obtained from Landwirtschaftliche Untersuchungs- und Forschungsanstalt.

The control validity criteria were fulfilled in most toxicity tests (online supplementary material Table S4). Exceptions were the high (>30%) coefficients of variation in juvenile numbers for lindane in LUFA 2.2 soil (46.5%), cyproconazole in OECD 2.5% soil (36.6%), and carbendazim in OECD 10% soil (41.9%). No significant differences were observed in survival and juvenile numbers between control and SC groups (Student’s *T*-test; *p* < .05), with the exception of reproduction in the carbendazim test in OECD 5% soil. Thus, the control and SC groups were grouped together for subsequent data analysis of the other tests, while for carbendazim reproduction in the OECD 5% soil, the SC control was used.

**Figure 1.**
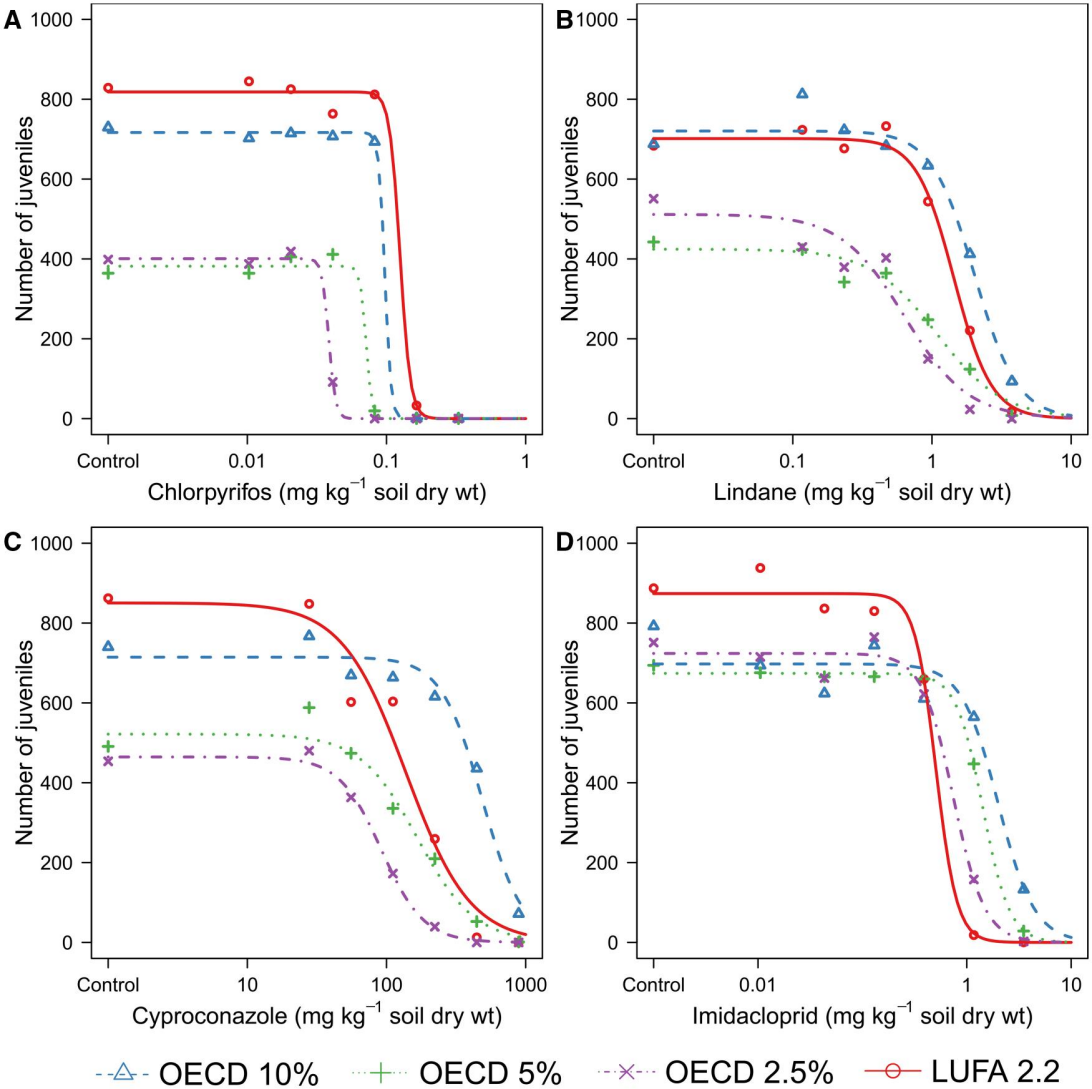
Dose–response relationships for the effects of chlorpyrifos (A), lindane (B), cyproconazole (C), and imidacloprid (D) on the reproduction of *Folsomia candida* exposed for 28 days in artificial (OECD 10%, OECD 5%, OECD 2.5%) and natural (LUFA 2.2) soils. Pesticide concentrations are corrected nominal concentrations based on average pesticide recoveries at the start of the tests. Symbols represent the average number of juveniles in the five replicates, lines show the fit of the three-parameter log-logistic dose–response models. OECD 10%: artificial soil containing 10% sphagnum peat; OECD 5%: artificial soil containing 5% sphagnum peat; OECD 2.5%: artificial soil containing 2.5% sphagnum peat; LUFA 2.2: natural reference soil obtained from the Landwirtschaftliche Untersuchungs- und Forschungsanstalt. OECD = Organisation of Economic Co-operation and Deveopment.

Median lethal concentration, EC50, and EC10 values for pesticide toxicity to the springtails in the four test soils are shown in Table 4 (based on corrected nominal values) and online supplementary material Table S5 (based on nominal values), and dose–response curves for survival and reproduction in online supplementary material Figure S1 and Figure 1, respectively. For carbendazim, no effects were observed on survival or reproduction at the highest test concentration, so the LC50 and EC50 values were >300 mg kg^−1 ^dry soil. Pesticide toxicity decreased in the order chlorpyrifos > lindane/imidacloprid > cyproconazole > carbendazim. For all pesticides, LC50 and EC50 values differed significantly between soils (likelihood ratio tests; *p* < .01), and toxicity in ASs increased in the order of OECD 10% > OECD 5% > OECD 2.5%. Pesticide toxicity in LUFA 2.2 soil did not show a clear pattern with regard to the other soils, with toxicity values being similar to OECD 10% soils for chlorpyrifos, between the OECD 10% and OECD 5% soils for lindane, between OECD 5% and OECD 2.5% soils for cyproconazole, and below those obtained in OECD 2.5% soil for imidacloprid.

**Table 4.** Values for the median lethal concentration (LC50), median effect concentration (EC50) and effect concentration for 10% (EC10) with corresponding 95% confidence intervals for the effect of pesticides (chlorpyrifos, lindane, cyproconazole, carbendazim, and imidacloprid) on the survival and reproduction of the springtail *Folsomia candida* in various artificial and natural soils with differing organic matter contents.^a^

Pesticide (mg kg^–1^ dry soil)	Soil			
	OECD 10%	OECD 5%	OECD 2.5%	LUFA 2.2
LC50				
Chlorpyrifos	0.25 (–1.4–1.9)	0.08 (0.07–0.09)	0.04 (0.04–0.05)	0.15 (0.04–0.26)
Lindane	>3.7^b^	3.8 (2.5–5.0)	2.3 (1.9–2.6)	3.8 (3.4–4.2)
Cyproconazole	>890^c^	634 (553–715)	284 (252–316)	666 (–133–1465)
Carbendazim^d^	>300	>300	>300	>300
Imidacloprid	12 (5.6–18)	6.4 (4.3–8.5)	5.3 (4.3–6.2)	1.3 (0.97–1.5)
EC50				
Chlorpyrifos	0.10 (–0.54–0.73)	0.07 (–0.04–0.19)	0.04 (0–0.07)	0.13 (0.08–0.17)
Lindane	2.0 (1.7–2.4)	1.1 (0.64–1.5)	0.63 (0.35–0.91)	1.4 (1.2–1.7)
Cyproconazole	488 (403–573)	167 (118–217)	92 (66–117)	138 (106–170)
Carbendazim^d^	>300	>300	>300	>300
Imidacloprid	2.0 (1.6–2.4)	1.4 (1.2–1.7)	0.75 (0.60–0.89)	0.51 (0.40–0.61)
EC10				
Chlorpyrifos	0.09 (–0.11–0.29)	0.06 (–0.12–0.25)	0.03 (–0.06–0.12)	0.10 (0.04–0.17)
Lindane	0.93 (0.54–1.31)	0.33 (–0.03–0.68)	0.20 (–0.07–0.47)	0.73 (0.50–0.96)
Cyproconazole	231 (115–348)	59 (25–93)	42 (18–67)	43 (20–66)
Carbendazim^d^	>300	>300	>300	>300
Imidacloprid	0.82 (0.47–1.2)	0.75 (0.46–1)	0.35 (0.22–0.48)	0.30 (0.23–0.38)

a All values are based on measured pesticide concentrations at the start of the tests.

b Survival was 90% ± 0.6 (SD) in control jars, and 74% ± 1.9 (SD) at the highest test concentration.

c Survival was 92% ± 1.2 (SD) in control jars, and 84% ± 1.9 (SD) at the highest test concentration.

d Nominal test concentrations.

*Note*. OECD 10%: artificial soil containing 10% sphagnum peat; OECD 5%: artificial soil containing 5% sphagnum peat; OECD 2.5%: artificial soil containing 2.5% sphagnum peat; LUFA 2.2: natural reference soil obtained from Landwirtschaftliche Untersuchungs- und Forschungsanstalt.

LC50 = Median lethal concentration.

EC50 = Median effect concentration reducing juveniles produced by 50% compared to the control.

EC10 = Effect concentration reducing juveniles produced by 10% compared to the control; OECD = Organisation for Economic Co-operation and Development.

**Figure 2.**
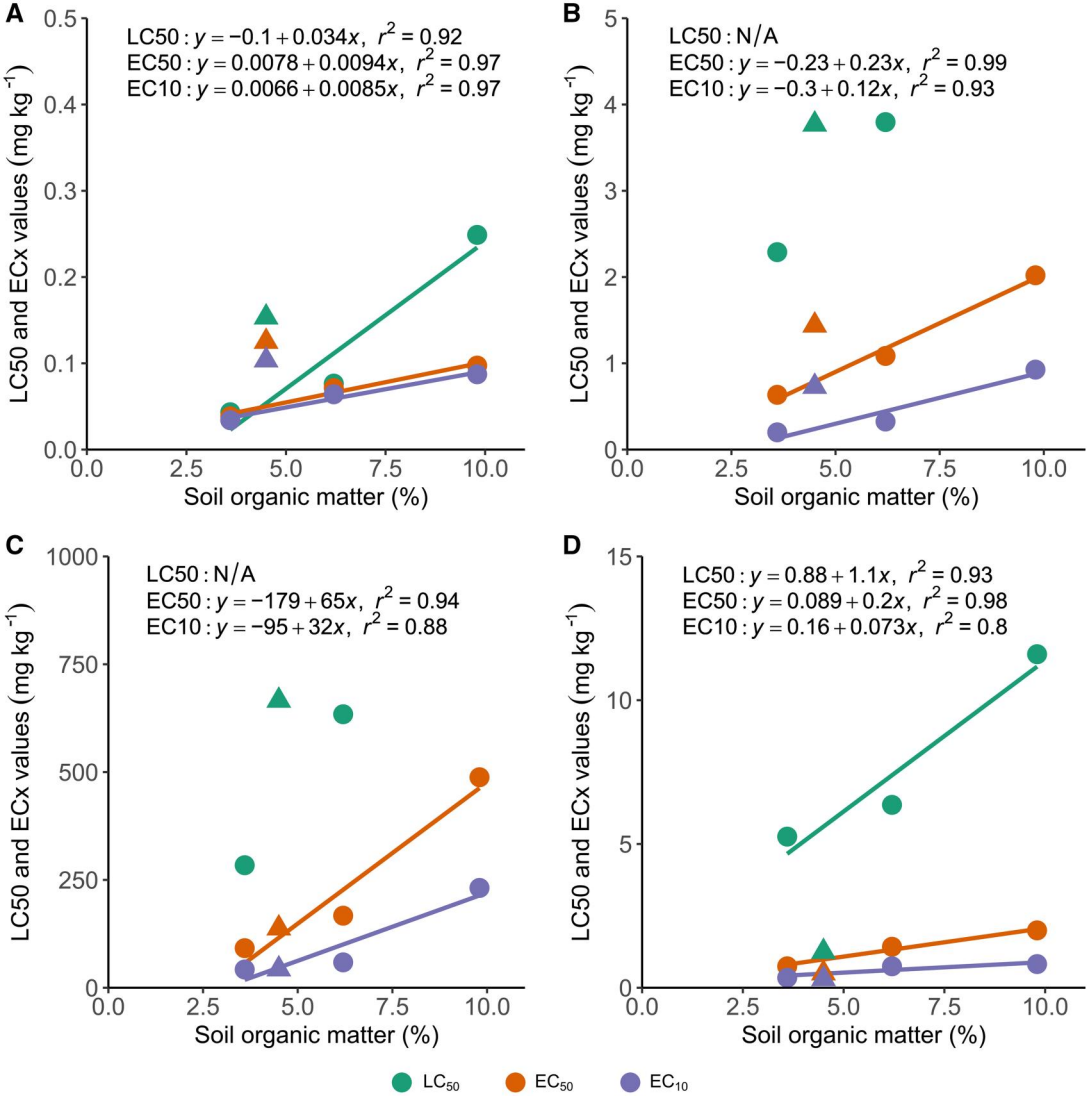
Relationships between pesticide toxicity (median lethal concentration [LC50], median effect concentration [EC50], and EC10 values) to *Folsomia candida* and soil organic matter (OM) content for (A) chlorpyrifos, (B) lindane, (C) cyproconazole, and (D) imidacloprid. Circles are data obtained for artificial soils, and triangles for LUFA 2.2 soil. Lines and equations show the linear regressions for the toxicity–OM relationships for the artificial soils only (LUFA 2.2 excluded). No regression analyses were performed for the lindane and cyproconazole LC50 data, because the LC50 values in artificial soil containing 10% peat could not be determined. LUFA 2.2: Natural reference soil obtained from the Landwirtschaftliche Untersuchungs- und Forschungsanstalt.

The relationships between pesticide toxicity to *F. candida* and soil OM content in ASs are shown in Figure 2. Regressions between LC50 and soil OM content were good for chlorpyrifos (Figure 2A) and imidacloprid (Figure 2D), with *r*^2^ values of 0.92 and 0.93, respectively, but could not be quantified for lindane and cyproconazole, because no LC50 values could be determined in the OECD 10% soil. For EC50 and EC10 values, the correlations were similar, with *r*^2^ values ranging between 0.92 and 0.99 for EC50 values, and between 0.88 and 0.97 for EC10 values. The toxicity data obtained in LUFA 2.2 soil did not correspond well with the toxicity–OM relationships constructed from the AS data, except for cyproconazole (Figure 2C). For chlorpyrifos and lindane, both lethal and sublethal toxicity in LUFA 2.2 soil was lower than expected based on the regressions (Figure 2A and B), while imidacloprid toxicity was higher (Figure 2D).

The regression equations from Figure 2 were used to assess the suitability of the additional CF of 2 by calculating the toxicity in soils containing 10% and 5% OM (Table 5). The difference in model-estimated toxicity between these soils ranged between 1.85–3.23 and 1.67–3.46, for EC50 and EC10 values, respectively. No clear patterns were evident with regard to pesticide lipophilicity and toxicity–OM relationships.

**Table 5.** Model-estimated median lethal concentration (LC50), median effect concentration (EC50), and 10% effect concentration (EC10) values for the toxicity (mg kg^−1^ dry soil) of pesticides to the springtail *Folsomia candida* in artificial soils with high (10.0%) and low (5.0%) soil organic matter (OM) contents.^a^

Pesticide	Endpoint	Regression equation	*r^2^*	Estimated LC50, EC50 or EC10 (mg kg^–1^) value		Toxicity ratio
				OM = 10.0%	OM = 5.0%	
Chlorpyrifos	LC50	*y* = –0.10 + 0.034x	0.92	0.24	0.07	3.43
	EC50	*y* = 0.008 + 0.009x	0.97	0.10	0.05	1.85
	EC10	*y* = 0.007 + 0.009x	0.97	0.10	0.05	1.87
Lindane	LC50	–	–	–	–	–
	EC50	*y* = –0.23 + 0.23x	0.99	2.07	0.92	2.25
	EC10	*y* = –0.30 + 0.12x	0.93	1.50	0.90	1.67
Cyproconazole	LC50	–	–	–	–	–
	EC50	*y* = –179 + 65x	0.94	471	146	3.23
	EC10	*y* = –95 + 32x	0.88	225	65	3.46
Imidacloprid	LC50	*y* = 0.88 + 1.1x	0.93	10.1	4.62	2.19
	EC50	*y* = 0.089 + 0.2x	0.98	2.09	1.09	1.92
	EC10	*y* = 0.16 + 0.073x	0.80	0.89	0.53	1.70

a LC50, EC50, and EC10 values were calculated using the regression equations for toxicity and OM content shown in Figure 2.

*Note*. OM: organic matter; LC50: median lethal concentration; EC50: median effect concentration reducing juveniles produced by 50% compared to the control; EC10: effect concentration reducing juveniles produced by 10% compared to the control.

To identify if relationships between pesticide toxicity and soil OM content differ between springtails and earthworms, the ratios between the model-estimated EC50 values in high and low OM ASs were compared with data generated by Van Hall et al. (2024; Table 6) in which earthworms (*E. andrei*) were exposed to the same pesticides in the same soils. The EC50 values were selected for comparison because reproduction is the endpoint currently used in ERA. Pesticide toxicity correlated strongly with soil OM content for both springtails and earthworms (*r*^2^ ≥ 0.82). For chlorpyrifos and lindane, the ratios between model-estimated toxicity in high and low OM soils were similar between species and close to the additional CF of 2 (ratios between 1.85 and 2.25). For cyproconazole, soil OM content seemed to influence toxicity approximately a factor of 1.5 more for *F. candida* than for *E. andrei*, with toxicity ratios being 3.05 and 1.94, respectively. The opposite was seen for imidacloprid, where toxicity ratios also differed by a factor of 1.5, but soil OM content influenced the toxicity more for *E. andrei* than for *F. candida*, with ratios of 1.92 and 3.05, respectively.

**Table 6.** Model-estimated median effect concentrations (EC50) values for the toxicity (mg kg^−1^ dry soil) of pesticides to the earthworm *Eisenia andrei* in artificial soils with high (10.0%) and low (5.0%) soil organic matter (OM) contents.

Pesticide	Regression equation	*r^2^*	Model-estimated toxicity (mg kg^–1^)		Toxicity ratio
			OM = 10.0%	OM = 5.0%	
Chlorpyrifos	*y* = –3.0 + 8.1x	0.96	78	37.5	2.08
Lindane	*y* = –4.4 + 4.5x	0.95	40.6	18.1	2.24
Cyproconazole	*y* = –11.7 + 9.1x	0.97	79	33.5	2.35
Imidacloprid	*y* = 0.72 + 0.26x	0.82	1.88	0.58	3.21

*Note*. Data originates from Van Hall et al. (2024), in which earthworms were exposed to the same pesticides in the same soils as used in this study.

## Discussion

The current study investigated the influence of soil OM content on pesticide toxicity to springtails by exposing *F. candida* to five pesticides in three ASs and one natural soil, thereby generating a unique and comparable toxicity dataset. The quality criteria from OECD guideline 232 were met in almost all cases, and the obtained toxicity values corresponded well with values obtained in similar soils from existing literature (online supplementary material Table S6), indicating that the tests were successfully performed.

The toxicity data reported in online supplementary material Table S6 show some variation between studies, with up to a factor of 5 difference between LC50 or EC50 values for similar soils but most often smaller differences. This variation in toxicity values may be attributed to differences between laboratories, for example, in culture conditions, methods of preparing ASs and spiking soils, variations in exposure conditions, and the expression of effects based on nominal or measured exposure concentrations. In addition, for some compounds, difference in OM content is not the only factor causing differences in toxicity. Ogungbemi and Van Gestel (2018), for instance, noted that also clay content may affect toxicity of imidacloprid to springtails. For that reason, this study used ASs prepared in the same way and using the same materials to ensure they only differed in OM contents.

The sections below each address one of the questions posed in the introduction section and discuss the implications for the European ERA.

### Toxicity–OM relationships and suitability of the CF of 2

The first question of the present study was if toxicity–OM relationships could be quantified for springtails, and whether they supported the application of the CF of 2. The results from this study showed that for both lipophilic and non-lipophilic pesticides, strong correlations were obtained between toxicity and OM content in AS (Figure 2), resulting in significant differences in toxicity between the soils. For all pesticides, the lowest toxicity was seen in OECD 10% soil, followed by OECD 5% soil, while the highest toxicity was seen in OECD 2.5% soil (Table 4). From these results, it is clear that soil OM content in ASs significantly influences pesticide toxicity to *F. candida*, and that toxicity–OM relationships can be quantified for springtails.

To assess whether the toxicity–OM relationships support the use of the CF of 2 to springtails, the ratios between model-estimated pesticide toxicity in high (10%) and low (5%) OM ASs were calculated using the regression formulas. This was done because the intent of the CF of 2 is to account for differences in toxicity between ASs containing 10% peat, and natural agricultural soils containing <5% OM (European Food Safety Authority (EFSA), 2015). For chlorpyrifos, lindane, and imidacloprid, the ratios ranged between 1.85 and 2.25 for EC50 values and 1.67 and 1.87 for EC10 values (Table 5), which is similar to the CF of 2. For cyproconazole, the model-estimated toxicity ratios were higher, being approximately a factor of 3 for both endpoints. These results suggest that the additional CF of 2 may not always be conservative enough if high OM content ASs are used in testing, especially when taking into consideration that natural soils usually contain less than the 5% OM (EFSA, 2015) used to model the toxicity in this study.

However, in contrast to earthworm toxicity tests, ASs used in springtail tests typically contain only 5% sphagnum peat, which is closer to the OM content found in European agricultural soils. Toxicity endpoints obtained in OECD 5% and LUFA 2.2 soil were very similar for all three lipophilic pesticides (Table 4). Thus, although our study shows that the influence of soil OM content on pesticide toxicity to springtails may be greater than is currently accounted for by the CF of 2, the study also shows that the CF of 2 is overly conservative and unnecessary when using soils containing 5% peat, which is typically the case for standardized toxicity tests using springtails.

Besides the CF of 2, the first tier of ERA also includes an assessment factor of 5 through the TER. The TER of 5 is included to cover for uncertainties occurring when extrapolating toxicity data from laboratory studies to field studies (i.e., climatic factors, differences in sensitivities between naturally occurring species and test species, soil properties). Because the results from this study show that the CF of 2 may be unnecessary when using AS containing only 5% peat, and all toxicity ratios calculated in this study were < 3.5, it appears that the TER of 5 already sufficiently captures the differences in toxicity caused by soil properties. However, our study only tested one natural soil (LUFA 2.2), and results are not necessarily similar in all natural soils (see for instance Natal-da-Luz et al., 2024). Moreover, it is unclear whether the TER of 5 also sufficiently captures the differences in toxicity caused by the other aforementioned factors in combination with the influence of soil properties, and thus whether it provides a sufficiently conservative and accurate ERA. Thus, the calibration between the first tier of ERA and field studies should be further investigated as has previously been done for earthworms (Christl et al., 2016) for example.

### Differences in springtail and earthworm toxicity–OM relationships

The second question of this study was about potential differences in toxicity–OM relationships between springtails and earthworms. Springtails and earthworms occupy different ecological niches, interact with soils in different ways, and often have different sensitivities to chemicals. Earthworms are generally considered detritivores, feeding on detritus and OM in the soil, thereby facilitating litter decomposition and nutrient cycling (Curry & Schmidt, 2007). The role of springtails is more diverse, but many species (including *F. candida*) feed on microorganisms such as fungi, bacteria, and algae, thus influencing microbial populations and communities and soil nutrient cycling (Lakshmi et al. 2020). These differences in ecological niches may result in different pesticide exposure routes between taxonomic classes and species, ultimately resulting in species-specific toxicity–OM relationships. For instance, previous studies have shown that, in earthworms, pesticide exposure through the gut may be an important exposure route for highly lipophilic chemicals (Belfroid et al., 1995). Because springtails generally do not ingest large amounts of soil, the influence of this exposure route is expected to be smaller than in earthworms. Other factors such as the body size and lipid content of a species may also cause differences in toxicity–OM relationships (Li et al., 2024). Indeed, a literature study by Van Hall et al. (2023) showed indications for species-specific toxicity–OM relationships for phenmedipham, because toxicity–OM relationships seemed to differ between enchytraeids and *F. candida*.

In the present study, pesticide sensitivity was species-specific, with *F. candida* being more sensitive to chlorpyrifos (up to 770×), lindane (up to 22×), and imidacloprid (up to 2×), and *E. andrei* being more sensitive to cyproconazole (up to 6×) and carbendazim (>188×) based on EC50 values. Despite these large differences in sensitivity, the toxicity–OM relationships were quite similar for both species, although they did differ in some cases. For chlorpyrifos and lindane, the toxicity ratios between high and low OM soils were similar for both species, with the ratios being approximately 2. In contrast, the toxicity ratios differed between earthworms and springtails for cyproconazole (2.08 and 3.23, respectively) and imidacloprid (3.21 and 1.92, respectively). These results were unexpected, because chlorpyrifos and lindane were the most lipophilic substances tested, and consequently the exposure through the gut was expected to be more important for these chemicals. Therefore, the influence of soil OM content would have been expected to be more important for earthworms, which ingest the soil, than to springtails, which are expected to be mostly exposed through the pore water. Belfroid et al. (1995) stated that uptake through the gut is irrelevant in most cases, except for highly lipophilic chemicals (log K_ow_ > 5) and in soils containing high OM content (20%). Thus, it may be the case that in our study, the lipophilicity of the test compounds, as well as the OM content of the test soils, were too low for gut uptake to become a relevant exposure pathway.

In any case, our study showed that toxicity–OM relationships can be different between species. Whether exposure is still mostly through pore water, and animal behavior or morphology (i.e., body size, lipid content) explains the observed differences, or if other exposure routes such as ingestion cause the observed differences requires further investigation.

### Comparisons between natural and ASs

The present study shows that soil OM content in AS corresponded well with pesticide toxicity, but an additional question posed in this study was if these relationships translate to natural soils. Overall, the observed toxicity in LUFA 2.2 soil did not correspond well with the obtained toxicity–OM relationships for AS. For chlorpyrifos and lindane, toxicity was lower than expected based on the OM content, while toxicity was higher for imidacloprid (Figure 2). These results suggest that other soil properties than OM content also influenced pesticide toxicity. For instance, the influence of clay content and clay type on imidacloprid bioavailability and toxicity has been reported several times for springtails (Bandeira et al., 2020; Ogungbemi & Van Gestel, 2018), and was also seen for earthworms (Van Hall et al., 2024).

In contrast to the springtail toxicity data, chlorpyrifos and lindane toxicity in LUFA 2.2 soil did correspond well with toxicity–OM relationships for earthworms (Van Hall et al., 2024). The differences in pesticide concentrations used in both studies may have explained some of these discrepancies. For the earthworm tests, the highest concentrations were 1,000 and 500 mg kg^−1^ for chlorpyrifos and lindane, respectively, while they were only 0.4 and 2.5 mg kg^−1^ for the springtail tests. It may be that the sorption to LUFA 2.2 soil was initially stronger than to the AS, resulting in higher sorption at low concentrations of chlorpyrifos and lindane. When higher concentrations are tested, the “strong” sorption sites in LUFA 2.2 may become saturated, and sorption between the soils becomes similar. Such saturation may not apply to the AS, where sorption could be more linear and less dependent on concentration.

### Toxicity–OM relationships and pesticide lipophilicity

The final question of our study was whether pesticide lipophilicity drives toxicity–OM relationships. The primary motivation for this question is that the CF of 2 is applied to lipophilic pesticides (log K_ow_ > 2), but that the influence of soil OM content on toxicity could increase with pesticide lipophilicity, or that soil OM content could also influence the toxicity of less lipophilic pesticides (log K_ow_ of 1.5–2). The results showed the largest differences in toxicity between soils for lindane and cyproconazole, which were not the most lipophilic pesticides tested, and similar differences in toxicity for chlorpyrifos and imidacloprid, which had the highest and lowest log K_ow_, respectively (Table 5). Thus, overall the results showed no clear influence of pesticide lipophilicity on toxicity–OM relationships.

These results were unexpected, because pesticide lipophilicity is one of the most important factors determining the sorption behavior of a chemical (Delle Site, 2001), and is therefore an essential factor in determining the bioavailability and the subsequent toxicity of a chemical. This reasoning is also included in a scientific opinion published by EFSA in 2017, where it was proposed to use an OM and K_om_-dependent scaling factor, instead of the fixed CF of 2, to account for the influence of these properties on pore water concentrations (EFSA, 2017). This scaling factor would allow for comparisons between (artificial) soils with different OM contents, by taking into account the normalized soil adsorption coefficient (K_om_) of the test chemical, with larger correction factors being applied for compounds with higher K_om_ values.

Ultimately, the findings of the present study do not support this proposition, because no clear influence of pesticide lipophilicity (log K_ow_) was seen on toxicity–OM relationships. Because K_ow_ and K_om_ are highly correlated (Rodriguez-Cruz et al., 2006), this raises a question over the relevance of using K_om_ values to account for the toxicity–OM relationship. In addition to this, K_om_ values are usually averages derived from tests using six natural soils, and because the sorption capacity between the OM types found in LUFA 2.2 and the sphagnum peat may differ, the applicability to AS remains uncertain. Finally, the sorption capacity of ASs constructed by different laboratories may also differ by an order of magnitude (Bielská et al., 2017), further complicating the comparisons between sorption of AS with natural soils. Therefore, the weight applied to a scaling factor based on (measured) K_om_ values, as well as the suitability of the scaling factor, requires further investigation.

## Conclusions

Our study showed that pesticide toxicity to the springtail *F candida* strongly correlated with soil OM content in ASs, with a lower toxicity at higher soil OM content, but that this relationship does not necessarily translate to natural soils. Moreover, no clear patterns between pesticide toxicity and OM content were observed with regard to endpoints, test organisms, and lipophilicity. These results show that the CF of 2 currently applied in European ERA is poorly supported by experimental evidence, and therefore needs further investigation.

## Supplementary Material

vgae048_Supplementary_Data

## Data Availability

The raw data used in this study can be found on the research repository Zenodo at https://doi.org/10.5281/zenodo.13906993.
