# Supplementary material for: The influence of soil organic matter content on the toxicity of pesticides to the springtail Folsomia candida
Source: Environ Toxicol Chem. 2025 Jan 6;44(2):524–33. doi: 10.1093/etojnl/vgae048 (PMC11816275; doi:10.1093/etojnl/vgae048)
Supplement: vgae048_Supplementary_Data [file vgae048_supplementary_data.docx]

# Supplementary Information to:

**The Influence of Soil Organic Matter Content on the Toxicity of Pesticides to the Springtail *Folsomia candida***

Table S1. Nominal concentration ranges (mg kg^-1^ dry soil) of the five pesticides used in the springtail toxicity tests using four soils with varying organic matter contents. SC = Solvent (acetone) control.

| **Pesticide** | **Concentration range (mg kg^-1^ dry weight soil)** | | | | | | | | | |
| --- | --- | --- | --- | --- | --- | --- | --- | --- | --- | --- |
| Chlorpyrifos | 0.00 | SC | 0.013 | 0.025 | 0.05 | 0.1 | 0.2 | 0.4 | - | - |
| Lindane | 0.00 | SC | 0.08 | 0.16 | 0.31 | 0.63 | 1.25 | 2.5 | - | - |
| Cyproconazole | 0.00 | SC | 31.25 | 62.5 | 125 | 250 | 500 | 1000 | - | - |
| Carbendazim | 0.00 | SC | 0.14 | 0.41 | 1.23 | 3.70 | 11.1 | 33.3 | 100 | 300 |
| Imidacloprid | 0.00 | - | 0.01 | 0.04 | 0.12 | 0.37 | 1.11 | 3.33 | 10 | - |

**Table S2. Average (± SD) soil pH (0.01 M CaCl_2_) at the lowest and highest test concentrations (n = 2) at the start (t = 0) and end (t = 28) of springtail (*Folsomia candida*) toxicity tests with pesticides in different artificial (OECD) and natural (LUFA 2.2) soils.**

| **Soil** | **Pesticide** | **Lowest test concentration** | | **Highest test concentration** | |
| --- | --- | --- | --- | --- | --- |
|  |  | **t = 0** | **t = 28** | **t = 0** | **t = 28** |
| OECD 10% | Chlorpyrifos | 5.96 ± 0.04 | 6.00 ± 0.03 | 5.96 ± 0.01 | 6.00 ± 0.00 |
|  | Lindane | 5.94 ± 0.09 | 6.08 ± 0.01 | 5.95 ± 0.00 | 6.09 ± 0.00 |
|  | Cyproconazole | 5.97 ± 0.01 | 6.03 ± 0.01 | 5.99 ± 0.01 | 6.13 ± 0.04 |
|  | Imidacloprid | 6.00 ± 0.07 | 6.06 ± 0.02 | 6.09 ± 0.03 | 6.07 ± 0.03 |
|  |  |  |  |  |  |
| OECD 5% | Chlorpyrifos | 5.79 ± 0.03 | 6.19 ± 0.09 | 5.81 ± 0.04 | 6.06 ± 0.02 |
|  | Lindane | 5.76 ± 0.07 | 6.00 ± 0.06 | 5.77 ± 0.04 | 6.00 ± 0.01 |
|  | Cyproconazole | 5.82 ± 0.07 | 5.87 ± 0.04 | 5.82 ± 0.04 | 5.79 ± 0.02 |
|  | Imidacloprid | 5.85 ± 0.02 | 5.77 ± 0.02 | 5.94 ± 0.02 | 5.76 ± 0.01 |
|  |  |  |  |  |  |
| OECD 2.5% | Chlorpyrifos | 5.95 ± 0.03 | 6.52 ± 0.00 | 6.00 ± 0.02 | 6.38 ± 0.03 |
|  | Lindane | 5.86 ± 0.12 | 6.28 ± 0.02 | 5.89 ± 0.04 | 6.40 ± 0.02 |
|  | Cyproconazole | 5.94 ± 0.02 | 6.19 ± 0.06 | 5.82 ± 0.04 | 6.07 ± 0.03 |
|  | Imidacloprid | 5.83 ± 0.07 | 6.07 ± 0.02 | 6.00 ± 0.02 | 6.00 ± 0.00 |
|  |  |  |  |  |  |
| LUFA 2.2 | Chlorpyrifos | 5.57 ± 0.06 | 5.38 ± 0.07 | 5.57 ± 0.04 | 5.54 ± 0.11 |
|  | Lindane | 5.53 ± 0.03 | 5.44 ± 0.01 | 5.64 ± 0.01 | 5.43 ± 0.04 |
|  | Cyproconazole | 5.52 ± 0.00 | 5.49 ± 0.12 | 5.61 ± 0.01 | 6.18 ± 0.01 |
|  | Imidacloprid | 5.56 ± 0.03 | 5.31 ± 0.02 | 5.54 ± 0.02 | 5.30 ± 0.06 |

**Table S3. Nominal pesticide concentrations, measured pesticide concentrations, and pesticide recovery in the different artificial (OECD) and natural (LUFA 2.2) test soils.** Measurements were done at concentrations around the EC_50_ values, and concentations were corrected for the moisture content of the soil (which was 50% of the water holding capacity, WHC).

| **Pesticide** | **Soil** | **Concentration (mg kg^-1^)** | | | **Recovery (%)** | |
| --- | --- | --- | --- | --- | --- | --- |
|  |  | **Nominal** | **Measured** | | **(WHC corrected)** | |
|  |  |  | **t=0** | **t=28** | **t=0** | **t=28** |
| Chlorpyrifos | OECD 10% | 0.1 | 0.07 | 0.06 | 89.3 | 76.5 |
|  | OECD 5% | 0.1 | 0.07 | 0.08 | 83.9 | 95.9 |
|  | OECD 2.5% | 0.05 | 0.03 | 0.03 | 69.6 | 69.6 |
|  | LUFA 2.2 | 0.1 | 0.06 | 0.03 | 73.8 | 36.9 |
|  | LUFA 2.2 | 0.2 | 0.16 | 0.08 | 98.4 | 49.2 |
|  |  |  |  |  |  |  |
| Lindane | OECD 10% | 1.25 | 1.50 | 1.30 | 153 | 133 |
|  | OECD 5% | 0.63 | 0.78 | 0.60 | 150 | 115 |
|  | OECD 2.5% | 0.31 | 0.43 | 0.35 | 159 | 130 |
|  | OECD 2.5% | 0.63 | 0.79 | 0.69 | 147 | 128 |
|  | LUFA 2.2 | 0.63 | 0.71 | 0.60 | 140 | 118 |
|  | LUFA 2.2 | 1.25 | 1.50 | 1.30 | 148 | 128 |
|  |  |  |  |  |  |  |
| Cyproconazole | OECD 10% | 500 | 319 | 321 | 81.3 | 81.9 |
|  | OECD 5% | 125 | 96.8 | 83.5 | 92.9 | 80.1 |
|  | OECD 5% | 250 | 194 | 172 | 93.0 | 82.5 |
|  | OECD 2.5% | 125 | 88.6 | 42.7 | 82.3 | 79.3 |
|  | OECD 2.5% | 250 | 181 | 90 | 84.0 | 83.6 |
|  | LUFA 2.2 | 125 | 110 | 102 | 108 | 100 |
|  | LUFA 2.2 | 250 | 180 | 192 | 88.6 | 94.5 |
|  |  |  |  |  |  |  |
| Imidacloprid | OECD 10% | 1.11 | 1.0 | 1.1 | 115 | 126 |
|  | OECD 10% | 3.33 | 2.3 | 2.4 | 88.1 | 91.9 |
|  | OECD 5% | 1.11 | 1.0 | 1.0 | 108 | 108 |
|  | OECD 5% | 3.33 | 3.2 | 2.5 | 115 | 90.0 |
|  | OECD 2.5% | 0.37 | 0.33 | 0.27 | 104.5 | 84.7 |
|  | OECD 2.5% | 1.11 | 1.0 | 0.97 | 105 | 101 |
|  | LUFA 2.2 | 0.37 | 0.31 | 0.24 | 103 | 79.8 |
|  | LUFA 2.2 | 1.11 | 0.94 | 0.72 | 104 | 79.8 |

Table S4. Control performance of *Folsomia candida* in the toxicity tests with five pesticides and four different artificial (OECD) and natural (LUFA 2.2) soils.

| **Pesticide** | **Soil** | **Average (± sd; n=5) number of surviving adults** | **Average (± sd; n=5) number of juveniles** | **Coefficient of variation (%)** |
| --- | --- | --- | --- | --- |
| Chlorpyrifos | OECD 10% | 8.6 ± 1.1 | 698.6 ± 66.6 | 9.5 |
|  | OECD 5% | 8.4 ± 0.9 | 339.2 ± 75.9 | 14.8 |
|  | OECD 2.5% | 8.2 ± 0.4 | 401.8 ± 59.6 | 22.4 |
|  | LUFA 2.2 | 9.4 ± 0.5 | 864.6 ± 143.7 | 16.6 |
|  |  |  |  |  |
| Lindane | OECD 10% | 9.0 ± 0.7 | 659.2 ± 44.1 | 6.7 |
|  | OECD 5% | 9.0 ± 0.8 | 476.3 ± 52.6 | 20.6 |
|  | OECD 2.5% | 9.0 ± 0.7 | 550.2 ± 113.3 | 11.1 |
|  | LUFA 2.2 | 8.4 ± 1.5 | 583.0 ± 271.1 | 46.5 |
|  |  |  |  |  |
| Cyproconazole | OECD 10% | 9.2 ± 1.3 | 723.2 ± 149.5 | 20.7 |
|  | OECD 5% | 9.6 ± 0.5 | 465.8 ± 170.6 | 20.3 |
|  | OECD 2.5% | 9.4 ± 0.5 | 454.8 ± 92.5 | 36.6 |
|  | LUFA 2.2 | 9.6 ± 0.5 | 881.2 ± 252.3 | 28.6 |
|  |  |  |  |  |
| Carbendazim | OECD 10% | 7.8 ± 1.9 | 625.4 ± 159.1 | 41.9 |
|  | OECD 5% | 9.0 ± 1.2 | 770.0 ± 119.6 | 15.5 |
|  | OECD 2.5% | 9.0 ± 0.7 | 414.0 ± 105.0 | 25.4 |
|  | LUFA 2.2 | 8.8 ± 0.8 | 960.4 ± 255.4 | 21.8 |
|  |  |  |  |  |
| Imidacloprid | OECD 10% | 9.0 ± 0.7 | 792.2 ± 115.8 | 14.6 |
|  | OECD 5% | 9.4 ± 0.5 | 694.2 ± 150.5 | 5.2 |
|  | OECD 2.5% | 9.8 ± 0.5 | 751.2 ± 38.8 | 21.7 |
|  | LUFA 2.2 | 10.0 ± 0.0 | 887.2 ± 83.8 | 9.4 |
|  |  |  |  |  |
| **Quality criteria** |  | **≥ 8** | **≥ 100** | **≤ 30** |

Table S5. LC_50_ and EC_x_ values with corresponding 95% confidence intervals for the effect of the pesticides chlorpyrifos, lindane, cyproconazole, carbendazim and imidacloprid on the survival and reproduction of the springtail *Folsomia candida* in different artificial (OECD) and natural (LUFA 2.2) soils containing different organic matter contents. All values are based on nominal pesticide concentrations in the test soils.

| **Pesticide**  **(mg kg^-1^ dry soil)** | **Soil** | | | |
| --- | --- | --- | --- | --- |
|  | **OECD 10%** | **OECD 5%** | **OECD 2.5%** | **LUFA 2.2** |
| LC_50_ |  |  |  |  |
| Chlorpyrifos | 0.31 (-11 – 12) | 0.09 (0.08 – 0.11) | 0.05 (0.05 – 0.06) | 0.19 (0.05 – 0.32) |
| Lindane | >2.5^a^ | 2.5 (1.7 – 3.3) | 1.5 (1.3 – 2.8) | 2.5 (2.3 – 2.8) |
| Cyproconazole | >1000^b^ | 883 (–) | 268 (–) | 740 (-34 – 1515) |
| Carbendazim | >300 | >300 | >300 | >300 |
| Imidacloprid | 11 (5.2 – 17) | 6.0 (4.1 – 8.0) | 5.0 (4.1 – 5.9) | 1.2 (0.93 – 1.5) |
|  |  |  |  |  |
| EC_50_ |  |  |  |  |
| Chlorpyrifos | 0.12 (–) | 0.09 (–) | 0.05 (-0.02 – 0.12) | 0.15 (0.10 – 0.21) |
| Lindane | 1.3 (1.1 – 1.6) | 0.72 (0.43 – 1.0) | 0.42 (0.24 – 0.61) | 0.96 (0.81 – 1.1) |
| Cyproconazole | 548 (453 – 644) | 188 (132 – 243) | 103 (74 – 132) | 155 (119 – 191) |
| Carbendazim | >300 | >300 | >300 | >300 |
| Imidacloprid | 1.9 (1.5 – 2.3) | 1.4 (1.1 – 1.6) | 0.71 (0.57 – 0.85) | 0.48 (0.38 – 0.58) |
|  |  |  |  |  |
| EC_10_ |  |  |  |  |
| Chlorpyrifos | 0.11 (–) | 0.08 (–) | 0.04 (-0.13 – 0.21) | 0.13 (0.05 – 0.20) |
| Lindane | 0.62 (0.36 – 0.87) | 0.22 (-0.02 – 0.45) | 0.13 (-0.05 – 0.31) | 0.49 (0.33 – 0.64) |
| Cyproconazole | 260 (129 – 391) | 66 (28 – 104) | 48 (20 – 75) | 48 (23 – 74) |
| Carbendazim | >300 | >300 | >300 | >300 |
| Imidacloprid | 0.78 (0.44 – 1.1) | 0.71 (0.44 – 1.1) | 0.33 (0.21 – 0.46) | 0.29 (0.22 – 0.36) |

^a^ Survival was 90% ± 0.6 (SD) in control jars, and 74% ± 1.9 (SD) in highest test concentrations.

^b^ Survival was 92% ± 1.2 (SD) in control jars, and 84% ± 1.9 (SD) in highest test concentrations.


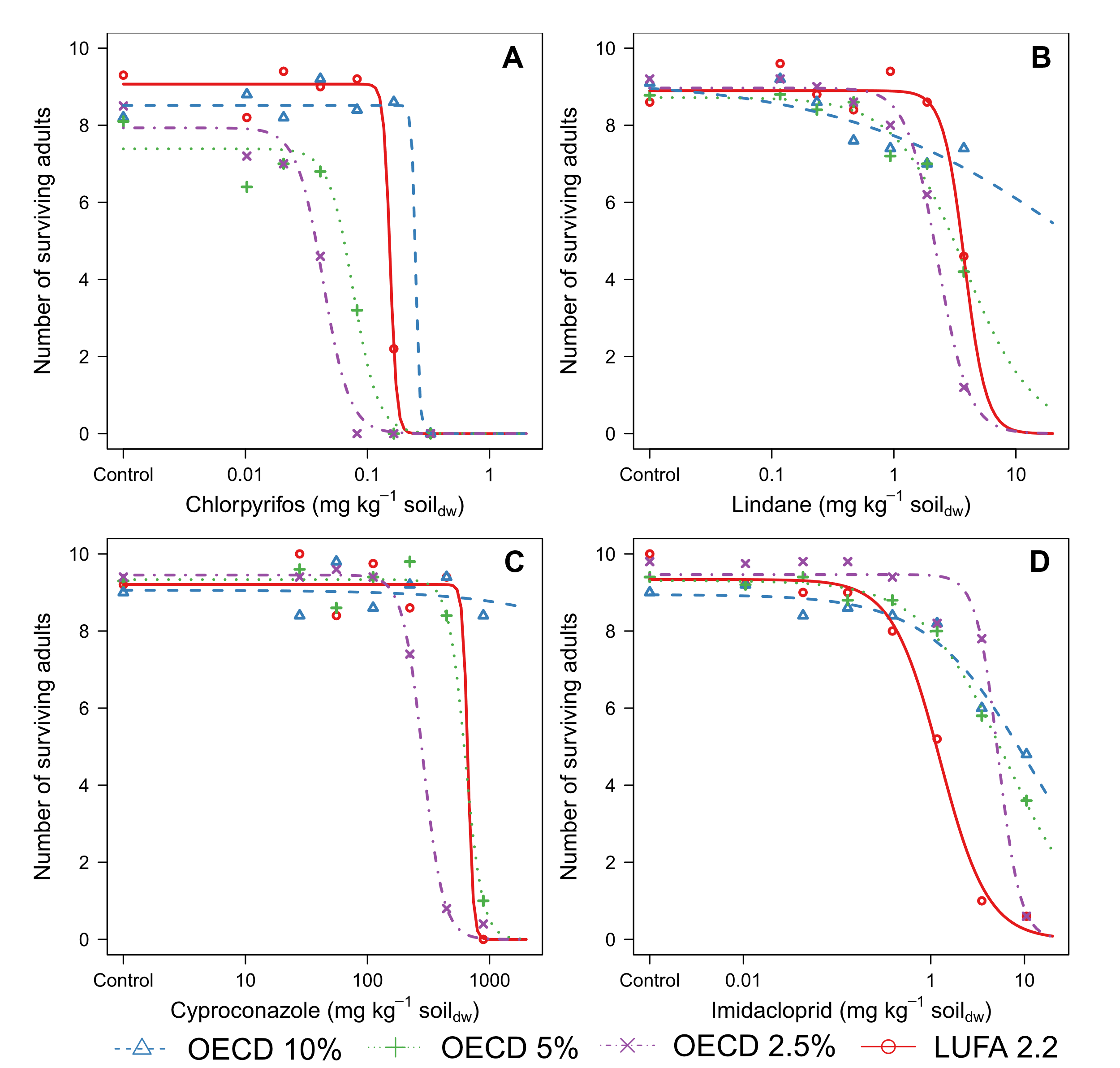


**Figure S1. Dose-response curves for the effects of chlorpyrifos (A), lindane (B), cyproconazole (C) and imidacloprid (D) on the survival of the springtail *Folsomia candida* exposed for 28 days in different artificial (OECD 10%, OECD 5%, OECD 2.5%) and natural (LUFA 2.2) soils.** Pesticide concentrations are corrected nominal concentrations based on average pesticide recoveries at the start of the tests (see Table 3). Symbols represent the average number of surviving adults in the five replicates, lines show the fit of the three-parameter log-logistic dose-response models.

**Table S6. Overview of toxicity data (LC_50_ and EC_50_) obtained from literature on the toxicity of the five pesticides to the springtail *Folsomia candida* in similar artificial (OECD) and natural (LUFA 2.2) soils as used in the current study.**

| **Pesticide** | **Reference** |  | **Toxicity value**  **(mg kg^-1^)** | |  |
| --- | --- | --- | --- | --- | --- |
|  |  | **Soil** | **LC_50_** | **EC_50_** | **Note** |
| Chlorpyrifos | Jaabiri Kamoun *et al.* (2017) | OECD 5% | 0.094 | 0.032 |  |
|  | Jegede *et al.* (2017) | OECD 5% | 0.04 | 0.031 |  |
|  | Selonen *et al.* (2023) | LUFA 2.2 | 0.13 | 0.10 |  |
|  | This study | OECD 5% | 0.08 | 0.07 |  |
|  |  | LUFA 2.2 | 0.15 | 0.13 |  |
|  |  |  |  |  |  |
| Lindane | Lock *et al.* (2002) | OECD 10% | 2.21 | 0.189 | 14-day LC_50_ |
|  | Amorim *et al.* (2012) | LUFA 2.2 | - | 0.8 |  |
|  | This study | OECD 10% | - | 2.0 |  |
|  | This study | LUFA 2.2 | 3.8 | 1.4 |  |
|  |  |  |  |  |  |
| Cyproconazole | Fernandes *et al.* (2023) | LUFA 2.2 | >1000 | 133 |  |
|  | This study | LUFA 2.2 | 665 | 138 |  |
|  |  |  |  |  |  |
| Carbendazim | IPCS InChem document | OECD 5 or 10% | - | >1000 |  |
|  | This study | OECD 5 + 10% | - | >300 |  |
|  |  |  |  |  |  |
| Imidacloprid | Ogungbemi & van Gestel (2018) | OECD 10% | 1.63 | 2.07 | 33-days |
|  |  | OECD 5% | 0.98 | 0.63 | 33-days |
|  |  | LUFA 2.2 | 0.31 | 0.14 | 33-days |
|  | Van Gestel *et al.* (2017) | LUFA 2.2 | 0.44 | 0.29 |  |
|  | De Lima e Silva *et al.* (2017) | LUFA 2.2 | 0.47 | 0.26 |  |
|  | De Lima e Silva *et al.* (2020) | LUFA 2.2 | 0.55 | 0.25 |  |
|  | This study | OECD 10% | 12 | 2.0 |  |
|  |  | OECD 5% | 6.4 | 1.4 |  |
|  |  | LUFA 2.2 | 1.3 | 0.51 |  |

**References for toxicity data:**

Amorim, M. J. B., Pereira, C., Menezes-Oliveira, V. B., Campos, B., Soares, A. M. V. M., & Loureiro, S. (2012). Assessing single and joint effects of chemicals on the survival and reproduction of *Folsomia candida* (Collembola) in soil. *Environmental Pollution*, *160*, 145-152. https://doi.org/10.1016/j.envpol.2011.09.005

De Lima e Silva, C., Brennan, N., Brouwer, J. M., Commandeur, D., Verweij, R. A., & van Gestel, C. A. M. (2017). Comparative toxicity of imidacloprid and thiacloprid to different species of soil invertebrates. *Ecotoxicology*, *26*, 555-564. https://doi.org/10.1007/s10646-017-1790-7

De Lima e Silva, C., De Rooij, W., Verweij, R. A., & Van Gestel, C. A. M. (2020). Toxicity in neonicotinoids to *Folsomia candida* and *Eisenia andrei*. *Environmental Toxicology and Chemistry*, *39*(3), 548-555. https://doi.org/10.1002/etc.4634

[IPCS] International Programme on Chemical Safety (1995). Carbendazim (Pesticide residues in food: 1995 evaluations Part II Toxicological & Environmental). *InChem document.* URL: https://inchem.org/documents/jmpr/jmpmono/v95pr19.htm

Jaabiri Kamoun, I., Jegede, O. O., Owojori, O. J., Bouzid, J., Gargouri, R., & Römbke, J. (2018). Effects of deltamethrin, dimethoate, and chlorpyrifos on survival and reproduction of the collembolan *Folsomia candida* and the predatory mite *Hypoaspis aculeifer* in two African and two European soils. *Integrated Environmental Assessment and Management*, *14*(1), 92-104. https://doi.org/10.1002/ieam.1966

Jegede, O. O., Owojori, O. J., & Römbke, J. (2017). Temperature influences the toxicity of deltamethrin, chlorpyrifos and dimethoate to the predatory mite *Hypoaspis aculeifer* (Acari) and the springtail *Folsomia candida* (Collembola). *Ecotoxicology and Environmental Safety*, *140*, 214-221. https://doi.org/10.1016/j.ecoenv.2017.02.046

Lock, K., De Schamphelaere, K. A. C., & Janssen, C. R. (2002). The effect of lindane on terrestrial invertebrates. *Archives of Environmental Contamination and Toxicology*, *42*, 217-221. https://doi.org/10.1007/s00244-001-0009-2

Ogungbemi, A. O., & van Gestel, C. A. M. (2018). Extrapolation of imidacloprid toxicity between soils by exposing *Folsomia candida* in soil pore water. *Ecotoxicology*, *27*, 1107-1115. https://doi.org/10.1007/s10646-018-1965-x

Selonen, S., Kokalj, A. J., Benguedouar, H., Alavian Petroody, S. S., Dolar, A., Drobne, D., & van Gestel, C. A. M. (2023). Modulation of chlorpyrifos toxicity to soil arthropods by simultaneous exposure to polyester microfibers or tire particle microplastics. *Applied Soil Ecology*, *181*, 104657. https://doi.org/10.1016/j.apsoil.2022.104657

Van Gestel, C. A. M., De Lima E Silva, C., Lam, T., Koekkoek, J. C., Lamoree, M. H., & Verweij, R. A. (2017). Multigeneration toxicity of imidacloprid and thiacloprid to *Folsomia candida*. *Ecotoxicology*, *26*, 320-328. https://doi.org/10.1007/s10646-017-1765-8
